# Systematic analysis of the lysine acetylome reveals diverse functions of lysine acetylation in the oleaginous yeast *Yarrowia lipolytica*

**DOI:** 10.1186/s13568-017-0393-2

**Published:** 2017-05-12

**Authors:** Guangyuan Wang, Lizhong Guo, Wenxing Liang, Zhenming Chi, Lin Liu

**Affiliations:** 1 College of Life Sciences, Shandong Province Key Laboratory of Applied Mycology, Qingdao Agricultural University, Qingdao, 266109 China; 2 The Key Laboratory of Integrated Crop Pest Management of Shandong Province, College of Agronomy and Plant Protection, Qingdao Agricultural University, Qingdao, 266109 China; 3 College of Marine Life Sciences, Ocean University of China, Qingdao, 266100 China

**Keywords:** Lysine acetylation, Acetylproteome, *Yarrowia lipolytica*, Oleaginous yeast, Lipid biosynthesis

## Abstract

Lysine acetylation of proteins, a major post-translational modification, plays a critical regulatory role in almost every aspects in both eukaryotes and prokaryotes. *Yarrowia lipolytica*, an oleaginous yeast, is considered as a model for bio-oil production due to its ability to accumulate a large amount of lipids. However, the function of lysine acetylation in this organism is elusive. Here, we performed a global acetylproteome analysis of *Y. lipolytica* ACA-DC 50109. In total, 3163 lysine acetylation sites were identified in 1428 proteins, which account for 22.1% of the total proteins in the cell. Fifteen conserved acetylation motifs were detected. The acetylated proteins participate in a wide variety of biological processes. Notably, a total of 65 enzymes involved in lipid biosynthesis were found to be acetylated. The acetylation sites are distributed in almost every type of conserved domains in the multi-enzymatic complexes of fatty acid synthetases. The provided dataset probably illuminates the crucial role of reversible acetylation in oleaginous microorganisms, and serves as an important resource for exploring the physiological role of lysine acetylation in eukaryotes.

**Electronic supplementary material:**

The online version of this article (doi:10.1186/s13568-017-0393-2) contains supplementary material, which is available to authorized users.

## Introduction

Post-translational modifications (PTMs) play an important role in modulating diverse cellular processes, which can change protein functions by introducing new functional groups such as phospho, acetyl, ubiquityl and methyl groups (Huang et al. 2015; Santos et al. 2011). Among them, acetylation of lysine is a highly dynamic and reversibly regulated PTM, which occurs on either the α-amino group at the N-terminus of the protein or the ε-amino group on the side chain of lysine residues (Choudhary et al. 2009; Wang et al. 2010; Mischerikow and Heck 2011). Lysine acetylation was first discovered in histone proteins (Allfrey et al. 1964; Phillips 1963) and its role has been extensively investigated in regulating gene transcription by histone acetyltransferases (HATs) or histone deacetylases (HDACs) modifying the core histone tails (Lee and Workman 2007; Yang and Seto 2008). However, in addition to histones, many other proteins in nucleus, cytoplasm, mitochondria, and other cellular compartment, were also found to be acetylated (Spange et al. 2009), which were involved in regulating a wide variety of important cellular processes, such as enzymatic activity (Nambi et al. 2013; Starai and Escalante-Semerena 2004), protein interactions (Choudhary et al. 2009) and metabolic pathways (Wang et al. 2010).

In recent years, utilization of microbial oils produced by oleaginous yeasts as sources in large-scale operations presents a great industrial interest. These microbial oils have been considered as one of the most suitable substitute for biodiesel production. And biodiesel has been well documented to have many advantages over the conventional diesel because of its biodegradable, non-toxic, and essentially free of sulfur and aromatic components (Helwani et al. 2009). Although several oleaginous yeasts, including *Cryptococcus curvatus*, *Lipomyces starkeyi*, *Rhodosporidium toruloides*, *Trichosporon capitatum* and *Yarrowia lipolytica*, can accumulate lipids more than 20% of their dry cell biomass (Papanikolaou and Aggelis 2011), the regulatory mechanism of lipid biosynthesis in oleaginous yeasts is poorly studied. Advances in liquid chromatography–mass spectrometry (LC–MS/MS) and lysine-acetylated peptide immune precipitation enable lysine acetylation to be investigated on a proteomic scale (Mo et al. 2015). So far a large number of lysine-acetylated proteins have been proved to be involved in various cellular functions, especially in regulating central metabolic pathways. In *Salmonella enterica*, key enzymes controlling the direction of glycolysis versus gluconeogenesis and the branching between citrate cycle and glyoxylate bypass were all regulated by acetylation (Wang et al. 2010). In budding yeast *Saccharomyces cerevisiae*, 1059 acetylated proteins were identified, and a large proportion of acetylated proteins were implicated in the regulation of glycolysis/gluconeogenesis and amino acid metabolisms (Henriksen et al. 2012). We wondered whether lysine acetylation plays an important role in the regulation of the production of lipid biosynthesis in oleaginous yeast.

*Yarrowia lipolytica* ACA-DC 50109, an oleaginous yeast, could produce lipid almost 50% (w/w) of the cell dry weight (Wang et al. 2013, 2015). In this study, we performed the first investigation on the acetylproteome of this microorganism. In total, we identified 3163 lysine acetylation sites in 1428 proteins, which account for 22.1% of the total proteins in the cell. The acetylated proteins are associated with diverse biological functions and cellular processes. Fifteen conserved acetylation motifs were detected through bioinformatics analysis of the sequences surrounding the acetylation sites. In addition, many enzymes involved in lipid biosynthesis were acetylated. These results provide a systems-wide view of acetylproteome and represent a dataset for functional analysis of acetylated proteins in *Y. lipolytica*.

## Materials and methods

### Strains, media and culture

The oleaginous yeast *Y. lipolytica* strain ACA-DC 50109 (Collection Number 2E00680 at the Marine Microorganisms Culture Collection of China), was kindly provided by Dr. Seraphim Papanikolaou (Papanikolaou and Aggelis 2003), was grown in yeast peptone dextrose (YPD) (10.0 g/L yeast extract, 20.0 g/L bacto peptone, and 20.0 g/L glucose). The compositions of the medium for lipid production were 60.0 g/L glucose, 7.0 g/L KH_2_PO_4_, 2.5 g/L Na_2_HPO_4_, 1.5 g/L MgSO_4_·7H_2_O, 0.15 g/L CaCl_2_, 0.15 g/L FeCl_3_·6H_2_O, 0.02 g/L ZnSO_4_·7H_2_O, 0.06 g/L MnSO_4_·H_2_O, and 0.5 g/L yeast extract (Wang et al. 2012). The yeast strain was incubated and harvested according to the methods described (Wang et al. 2015). In brief, 2.0 mL of yeast cell suspension (2.5 × 10^8^ cells/mL) which had been pre-cultivated in YPD medium for 24 h were added to 50 mL of the lipid production medium. The culture was incubated on a rotary shaker at 180 rpm and 28 °C for 72 h. After that, cells in the culture broth were harvested by centrifugation (8000×*g* for 5 min) and washed three times with sterile distilled water.

### Lipid determination and observation of lipid particles in the yeast cells

The total lipids in the cells (1.0 g) were extracted according to the methods described (Folch et al. 1957). The extracted lipids were weighted and oil content per 100 g of cell dry weight was calculated. The lipid particles in the yeast cells were stained by Nile Red and observed according to the methods described (Wang et al. 2012).

### Protein extraction and trypsin digestion

After the yeast cells were grinded in liquid nitrogen, the protein in the sample was extracted according to the procedures described (Li et al. 2016; Liu et al. 2016). Protein concentration extracted above was determined using a 2-D Quant kit (GE Healthcare) according to the manufacturer’s instructions. For digestion, the protein solution was reduced with dithiothreitol (DTT) (5 mM) for 30 min at 56 °C and alkylated using iodoacetamide (IAA) (20 mM) at room temperature for 45 min in darkness. Finally, the resulting sample was digested using trypsin (Li et al. 2016; Liu et al. 2016).

### Affinity enrichment

Samples obtained as described in the previous section was separated into 6 fractions by high pH reverse-phase HPLC using an Agilent Systerm with a ZORBAX 300Extend-C18 column (5 μm particles, 4.6 mm ID, 250 mm length). Acetylated peptides in each fraction were enriched using anti acetyllysine antibody beads (PTM Biolabs, Cat. No. 104, Hangzhou, China). Finally, the resulting peptides were cleaned with C18 ZipTips (Millipore) followed by LC–MS/MS analysis.

### LC–MS/MS analysis

The acetylated peptides obtained above were dissolved in 0.1% formic acid (FA) and separated on a reversed-phase Acclaim Pep-Map RSLC C18 column (2 μm, 50 μm × 15 mm, 100 Å) (Thermo Scientific) by a gradient elution of 0.1% FA and 98% acetonitrile, with a flow rate of of 300 nL/min on an EASY-nLC 1000 UPLC system (Zhou et al. 2016). The resulting peptides were subjected to electrospray/nanospray ionization (ESI/NSI) and analyzed by MS/MS using Q Exactive™ Plus tandem mass spectrometry (Thermo Scientific). The electrospray voltage applied was 2.0 kV. The m/z scan range was 350–1800 for full scan, and intact peptides were detected in the Orbitrap at a resolution of 70,000. Peptides were then selected for MS/MS using NCE setting as 30 and the fragments were detected in the Orbitrap at a resolution of 17,500. A data-dependent procedure that alternated between one MS scan followed by 20 MS/MS scans with 15.0 s dynamic exclusion. Automatic gain control (AGC) was set at 5E4.

### Database search

MaxQuant with integrated Andromeda search engine (v.1.4.2) was employed to process the resulting MS/MS data (Cox and Mann 2008; Cox et al. 2009). Tandem mass spectra were searched against UniProt-*Y. lipolytica* database (6453 sequences) concatenated with reverse decoy database. Trypsin/P was specified as cleavage enzyme allowing up to 4 missing cleavages. The mass tolerance for precursor ions was set as 20 ppm in First search and 5 ppm in Main search, and the mass tolerance for fragment ions was set as 0.02 Da. Carbamidomethyl on Cys was specified as fixed modification and acetylation on Lys and oxidation on Met were specified as variable modifications. False discovery rate (FDR) was adjusted to <1% and the site localization probability was set as >0.75.

### Bioinformatics analyses

For functional annotations of all the acetylated proteins obtained in the previous section, enrichment analyses based on Gene Ontology (GO) (http://www.ebi.ac.uk/GOA) and Kyoto Encyclopedia of Genes and Genomes (KEGG) pathways were investigated (Kanehisa et al. 2004), respectively. Functional description of protein domains was annotated by InterProScan based on protein sequence alignment method, and the InterPro domain database was used (Hunter et al. 2009). Wolfpsort, a subcellular localization predication soft, was used to predict subcellular localization of the identified acetylated proteins (Horton et al. 2007). DAVID bioinformatics resources 6.7 was used to identify GO term, KEGG pathway and protein domain (Jiao et al. 2012). A two-tailed Fisher’s exact test was used to test specific annotation terms among members of resultant protein clusters. Any annotation categories with Fisher’s exact test *p* value <0.05 were considered strongly enriched. Motif-x software was employed to analyze the model of sequences constituted with amino acids in specific positions of acetyl-21-mers (10 amino acids upstream and downstream of the site) in all protein sequences (Chou and Schwartz 2011). Protein–protein interactions for the identified acetylated proteins were performed using Cytoscape software and the protein–protein interaction network was obtained from the STRING database according to the methods described previously (Shannon et al. 2003).

### SDS-PAGE and Western blotting

The proteins extracted above were determined by sodium dodecyl sulfate polyacrylamide gel electrophoresis (SDS-PAGE) and Western blotting. Briefly, 12% gel in SDS-PAGE was used to separate the proteins with different molecular weight and then all the proteins were electrotransferred onto a polyvinylidene difluoride (PVDF) membrane using a Bio-Rad Trans-Blot system. 5% skim milk powder was used to block the membranes in TBST buffer (20 mM Tris–HCl, 150 mM NaCl, 0.05% Tween 20) for 30–60 min at room temperature. After that, the PVDF membranes were incubated with pan anti-acetyl lysine antibody (PTM Biolabs, 101), anti-histone H2A.Z antibody (Abcam, ab4174) and anti-H2A.Z (acetyl K7) antibody (Abcam, ab214730) (1:1000, in TBS/5% skim milk powder) for 1 h at room temperature, respectively. After the PVDF membranes were washed 3 times with TBST buffer, the membranes were incubated with horseradish peroxidase (HRP) conjugated anti-rabbit IgG (1:5000 dilutions) for 1 h at room temperature. Last, an enhanced chemiluminescence (ECL) immunoblotting detection kit (Beyotime Biotechnology, Shanghai) was used for signal detection.

## Results

### Proteome-wide analysis of lysine acetylation sites and proteins in *Y. lipolytica*

**Fig. 1:**
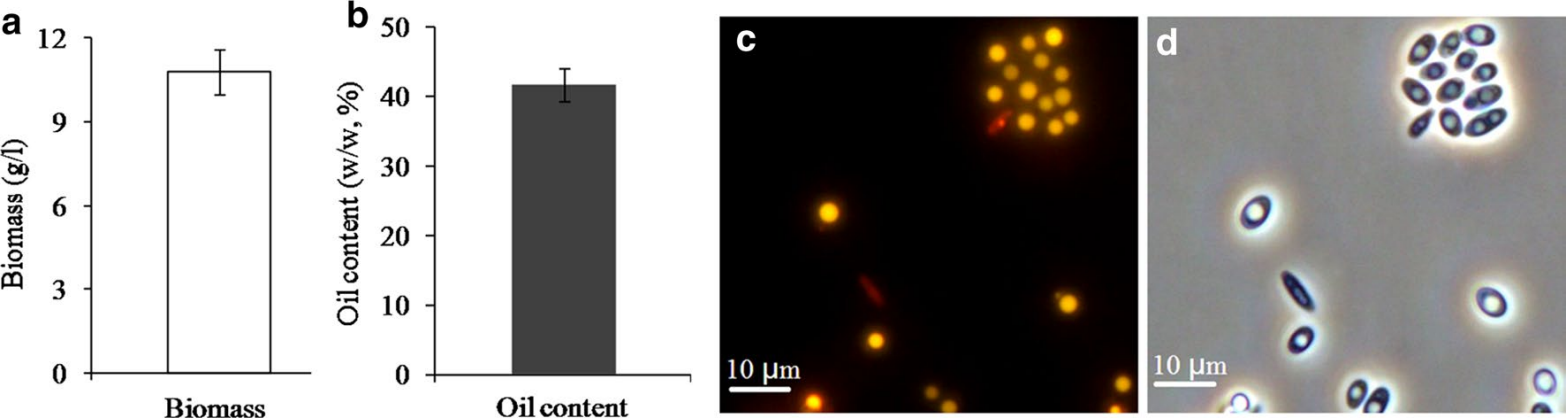
Cell mass, oil content and lipid particles of the yeast strain ACA-DC 50109. **a** Biomass of the yeast strain. Data are given as mean ± SD, n = 3. **b** Oil content of the yeast strain. Data are given as mean ± SD, n = 3. **c** Lipid particles of the yeast strain were taken under fluorescent microscope. **d** Lipid particles of the yeast strain were taken under phase microscope

**Fig. 2:**
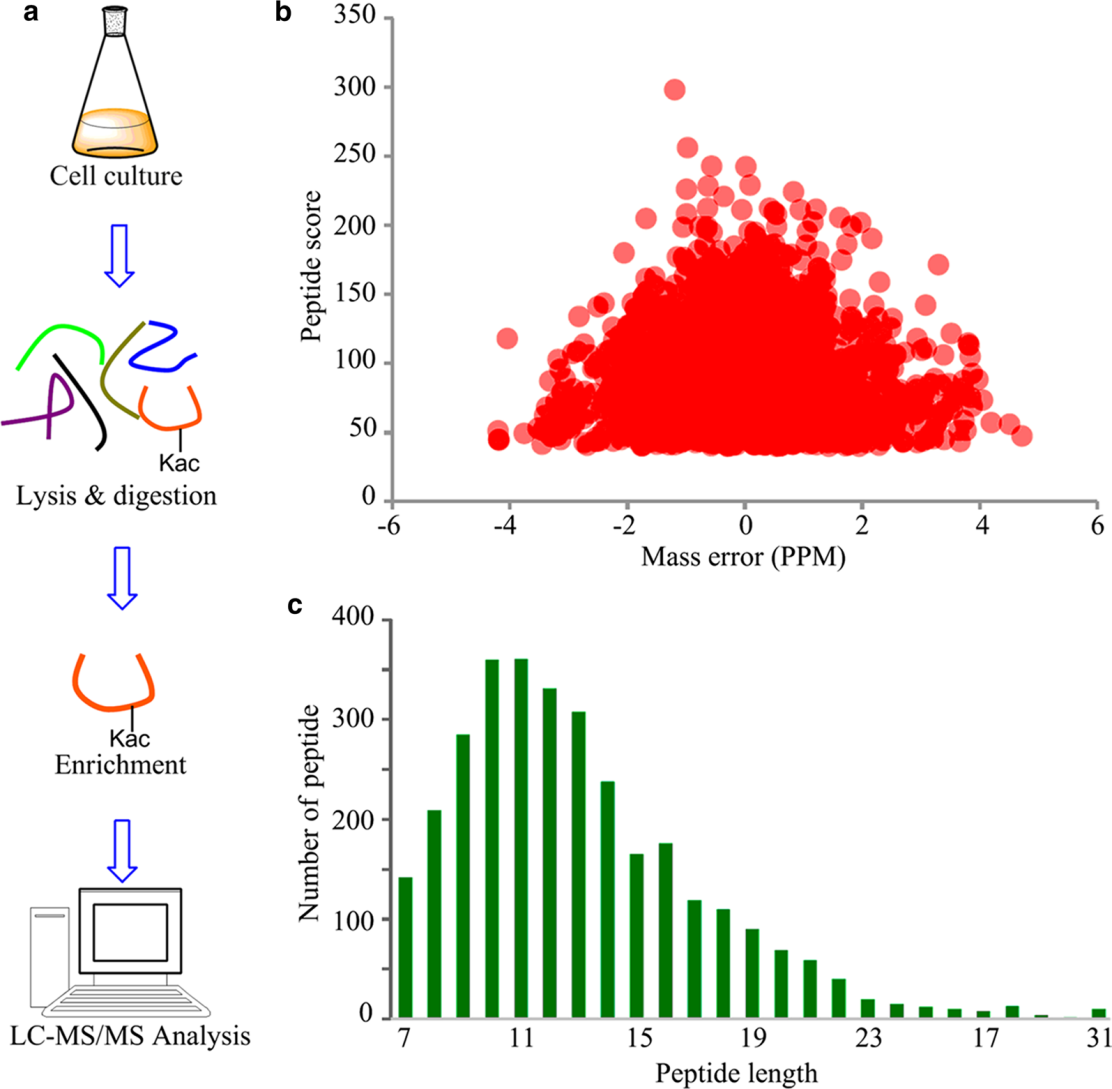
Proteome-wide identification of lysine acetylation sites in *Y. lipopytica*. **a** Overview of experimental procedures used in this study. **b** Mass error distribution of all identified peptides. **c** Peptide length distribution

*Yarrowia lipolytica* ACA-DC 50109 was cultured in the oil production medium. As shown in Fig. 1, 10.8 g/L of the dry cell mass (Fig. 1a), 41.6% (w/w) of lipid based on cell dry weight were obtained (Fig. 1b), respectively. And most of the cells contained big lipid bodies and were stained by Nile Red (Fig. 1c, d), indicating that ACA-DC 50109 could synthesize high-level lipid in the cells. To elucidate the regulatory functions of lysine acetylation in this oleaginous yeast, we performed a proteome-scale analysis of acetylated proteins and acetylated sites in *Y. lipolytica*. Overview of experimental procedures used in this study was shown in Fig. 2a. Proteins were extracted and digested with trypsin, and then total peptides were subjected to acetylated enrichment with anti-acetyllysine antibody beads and analyzed using high-resolution LC–MS/MS. To validate the MS data, the mass error of all the identified peptides were checked and the results showed that the distribution of mass error was near zero and most of them were less than 5 ppm (Fig. 2b), which means the mass accuracy of the MS data fits the requirement. The length of most peptides was distributed between 7 and 25, which agrees with the property of tryptic peptides (Fig. 2c), and means that sample preparation reaches the standard. Additional file 1: Figure S1 shows three examples of MS/MS spectra of acetylated peptides. As such, totally 3163 lysine acetylation sites that matched to 1428 unique proteins from the UniProt_*Y. lipolytica* database were identified (Additional file 2: Table S1). The mass spectrometry proteomics data have been deposited to the ProteomeXchange Consortium via the PRIDE partner repository with the dataset identifier PXD006272.

**Fig. 3:**
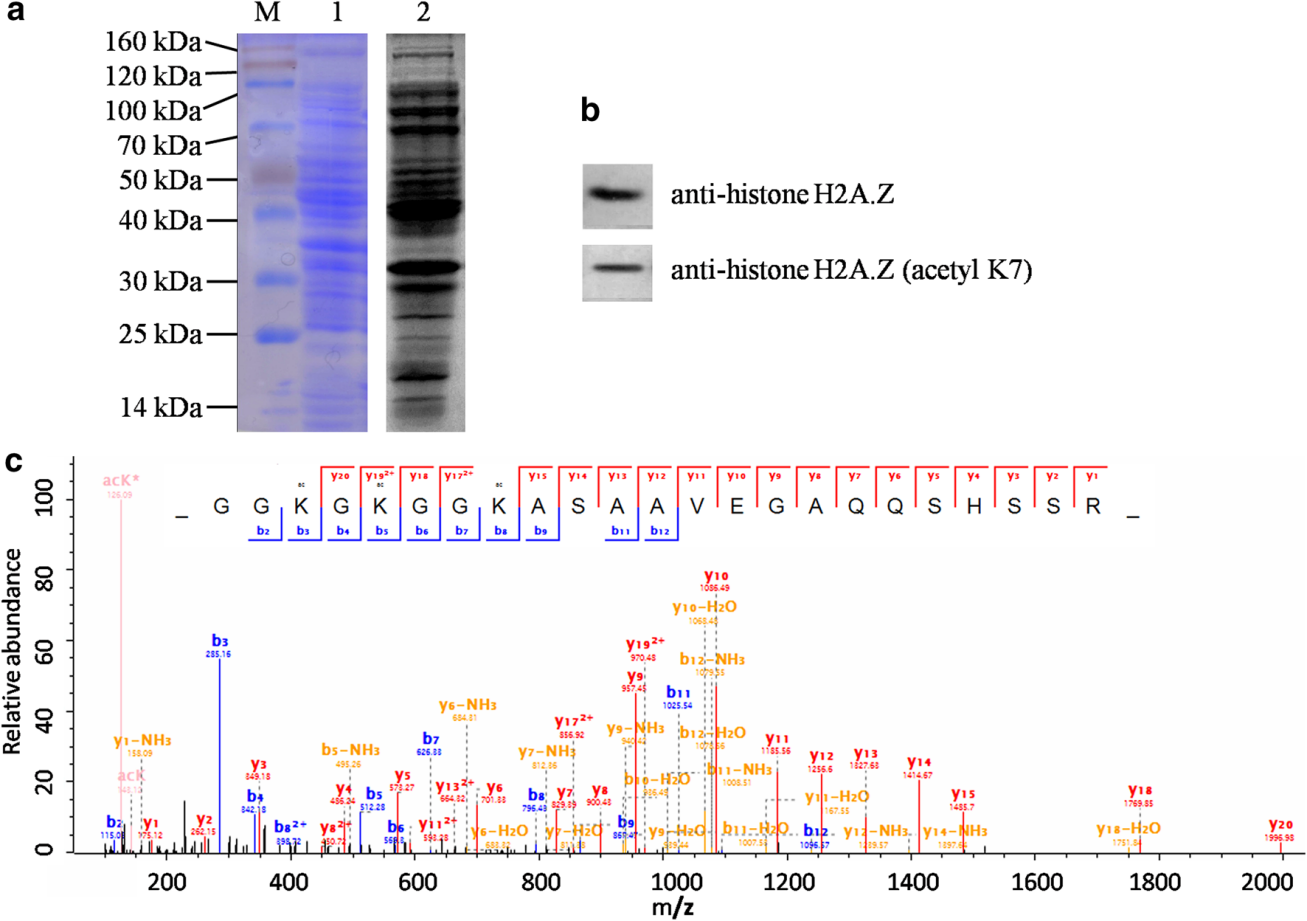
An overview of lysine acetylation in *Y. lipolytica* as analyzed by SDS-PAGE and Western blotting. **a** 100 μg of total protein was separated by SDS-PAGE (*1*) and the acetylated proteins were detected with pan anti-acetyl lysine antibody (*2*). **b** The Western blotting signal with anti-histone H2A.Z antibody and anti-H2A.Z (acetyl K7) antibody. **c** MS/MS spectra of acetylpeptide with an acetylation site at K7 of histone H2A.Z (Q6C341)

To validate the diversity of acetylated proteins in *Y. lipolytica*, we performed Western blotting analysis using pan anti-lysine acetylation antibodies. As shown in Fig. 3a line 2, multiple immunoblot signals with a wide range of molecular weights were observed, indicating that acetylated proteins was abundant in *Y. lipolytica*. According to the LC–MS/MS analysis, an acetylated site at K7 of histone H2A.Z (Q6C341) was obtained (Additional file 2: Table S1; Fig. 3c). In order to confirm this acetylated site, we further performed Western blotting analysis using anti-histone H2A.Z (acetyl K7) antibody. Consistent with the MS/MS spectra of histone H2A.Z (K7) (Fig. 3c), a legible band was observed after ECL immunoblotting detection (Fig. 3b), indicating the mass spectrometry proteomics data are reliable.

### Distribution and motif analysis of lysine acetylation sites

**Fig. 4:**
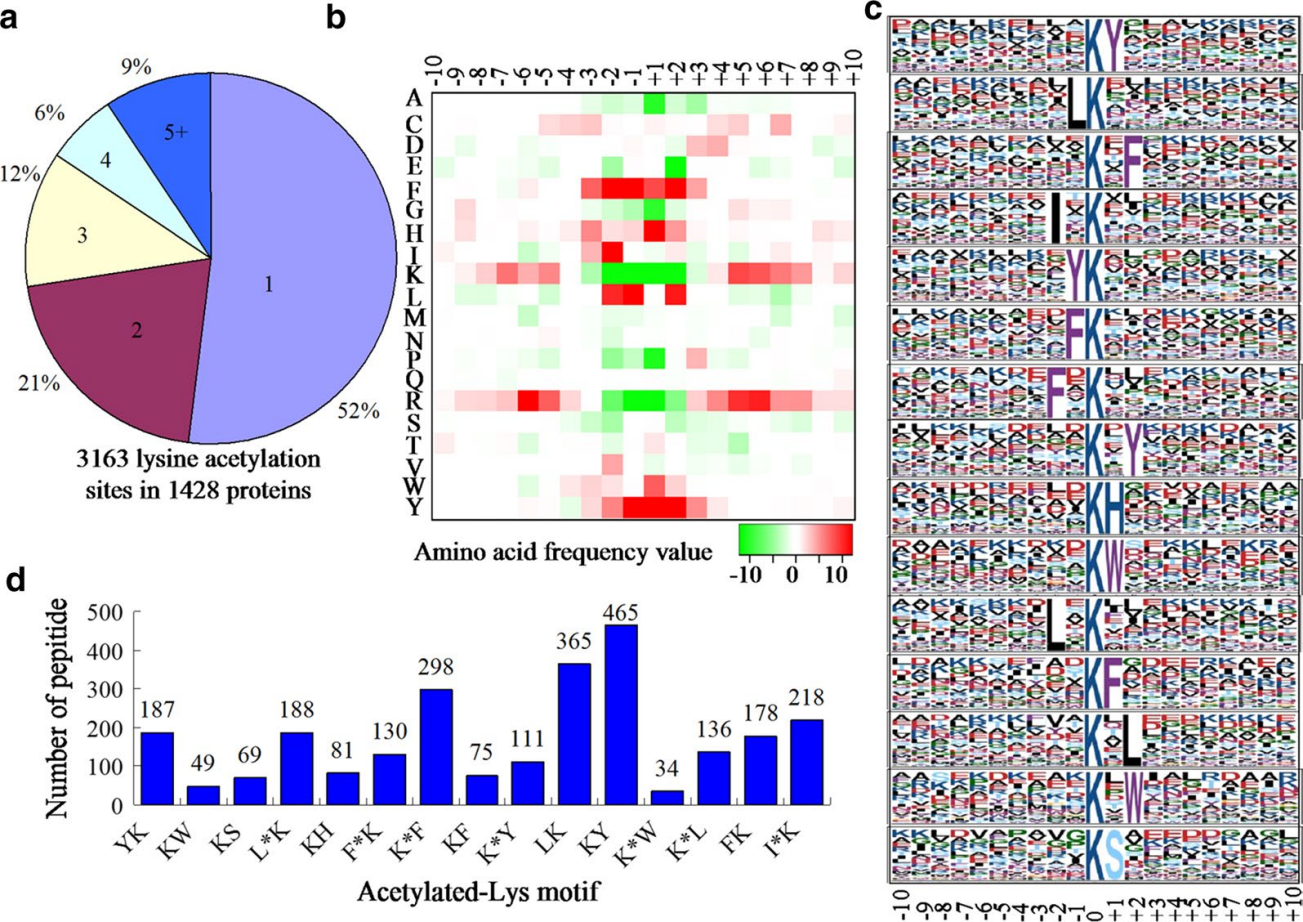
Distribution and motif analysis of lysine acetylation sites. **a** Pie chart illustrating the number and percentage of lysine acetylation sites per protein. **b** Heat map of the amino acid compositions around the lysine acetylation sites showing the frequency of different types of amino acids surrounding this residue. **c** Sequence probability logos of significantly enriched acetylation site motifs for ±10 amino acids around the lysine acetylation sites. **d** Number of identified peptides contained in each conserved motif

In order to evaluate the distribution of acetylation sites in the acetylated proteins of *Y. lipolytica*, we calculated the numbers of identified modification sites per protein. The results showed that 52% of proteins contained only one acetylation site, and the percentage of proteins with two, three, four and five or more modification sites were 21, 12, 6, and 9%, respectively (Fig. 4a). The heat map of different types of amino acids from −10 to +10 surrounding the acetylation sites was investigated. The frequency of phenylalanine (F), isoleucine (I), leucine (L) and tyrosine (Y) in positions −2 to +2 was highest, while the occurrence of lysine (K), glutamic acid (E) and arginine (R) was lowest (Fig. 4b).

To further assess the character of acetylated proteins in *Y. lipolytica*, the sequence motifs in all of the identified acetylated peptides were analyzed using the motif-x program. Totally, 15 conserved sequences were identified (Fig. 4c), which exhibited different abundances (Fig. 4d). Importantly, although most of the motifs have been found in other microorganisms (Huang et al. 2015; Li et al. 2016; Liao et al. 2014; Liu et al. 2016; Lv et al. 2016; Pan et al. 2014; Xie et al. 2015), the significantly conserved motif, K^ac^XW (K^ac^ represents acetylated lysine and X represents a random amino acid residue) in *Y. lipolytica*, were identified for the first time. A survey of these motifs indicated that four distinct types of residues were found around the acetylated lysine: a residue with hydrophobic side chain groups, such as L and I; a residue with aromatic groups, including Y, F and tryptophan (W); a positively charged residue, histidine (H); and a polar amino acid residue, serine (S) (Fig. 4c).

### Secondary structure analysis of acetylated proteins

**Fig. 5:**
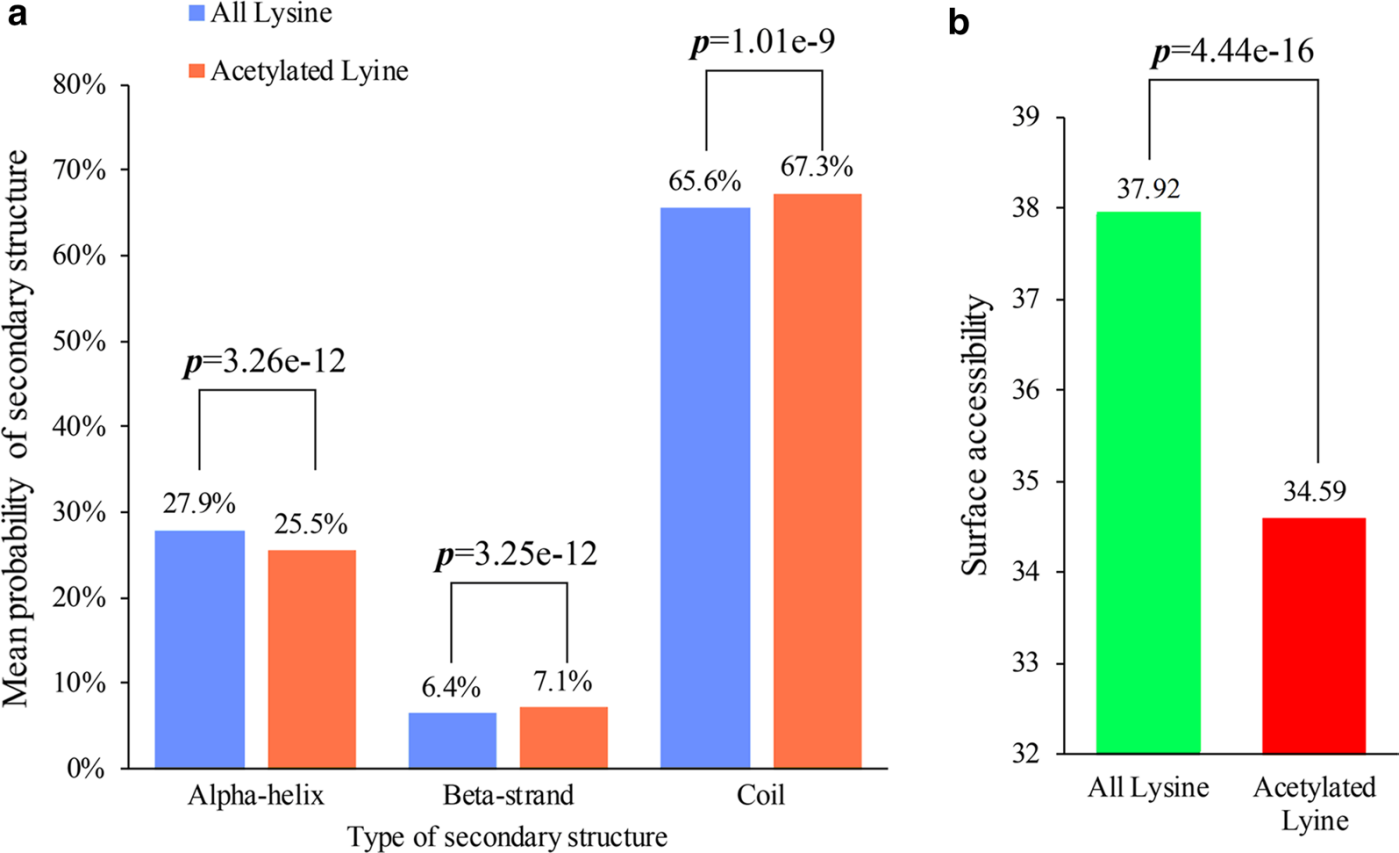
Secondary structure analysis of acetylated proteins. **a** Probabilities of lysine acetylation in the structures of alpha-helix, beta-strand and coil. **b** Predicted surface accessibility of acetylation sites

To elucidate the relationship between the acetylated sites and protein structure, we performed a structural analysis of all the acetylated proteins in *Y. lipolytica* (Fig. 5). As shown in Fig. 5a, 32.6% (1031) of the acetylation sites was located at regions with ordered secondary structures. Among them, 25.5% (806) was located in α-helix and 7.1% (225) in beta-strand (Fig. 5a). In addition, 67.3% (2132) of the acetylation sites was distributed in the disordered structures of proteins (Fig. 5a). Although the distribution pattern of all lysine was similar to that of acetylated lysine, the difference between them was significant (*p* < 0.001) (Fig. 5a), suggesting an interesting relationship between lysine acetylation and secondary structures. Surface accessibility of the acetylated lysine sites was also analyzed. The results in Fig. 5b showed that, compared with 37.92% of all lysine residues, 34.59% of the modified sites (*p* = 4.44e−16) was located on the protein surface. Thus, surface property of proteins is likely be changed by lysine acetylation in the oleaginous yeast *Y. lipolytica*.

### Functional annotation and cellular localization of acetylated proteins

**Fig. 6:**
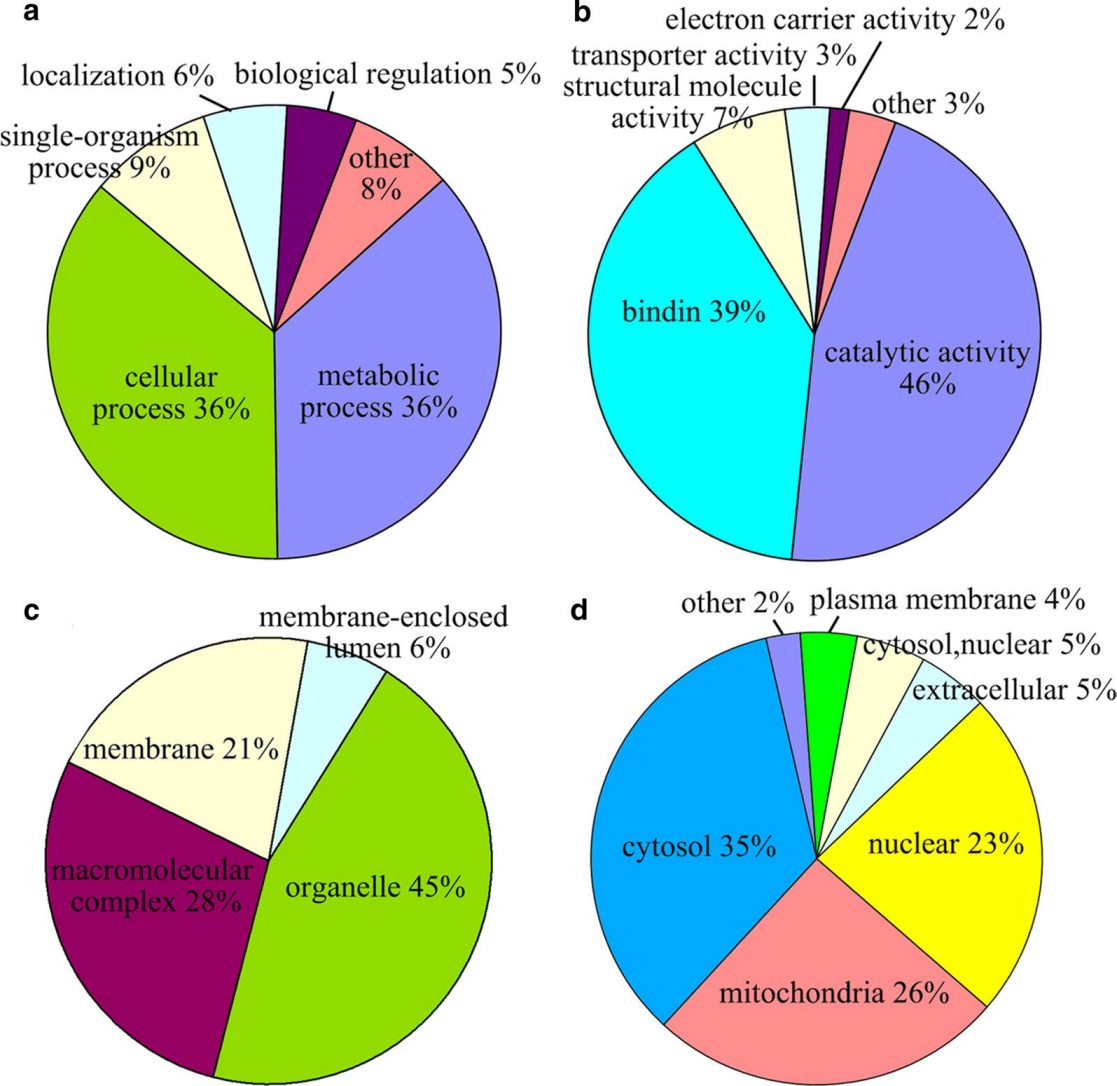
Pie charts showing the functional classification of acetylated proteins in *Y. lipolytica*. **a** Classification of the acetylated proteins based on biological process. **b** Classification of the acetylated proteins based on molecular function. **c** Classification of the acetylated proteins based on cellular component. **d** Subcellular localization of the acetylated proteins

To better understand the lysine acetylome of *Y. lipolytica*, the GO functional classification of all the acetylated proteins based on their biological process, molecular function and cellular component was investigated (Fig. 6a–c). The GO analysis indicated that the acetylated proteins were distributed broadly in terms of their biological processes. Among them, the largest group is proteins associated with metabolic processes (36%) and cellular process (36%) (Fig. 6a). According to the GO molecular function category, the largest proportion of identified proteins were associated with catalytic activity (46%) (Fig. 6b), and the second largest group of acetylated proteins was found to be related to binding (39%) (Fig. 6b). The results of cellular component analysis showed that the acetylated proteins identified were distributed in organelles (45%), macromolecular complex (28%), membrane (21%) and membrane-enclosed lumen (6%) (Fig. 6c). As shown in Fig. 6d, most of the acetylated proteins in *Y. lipolytica* were distributed in the cytosol (35%), mitochondria (26%) and nucleus (23%), as determined by subcellular localization analysis. Taken together, these data demonstrates that the lysine acetylated proteins in *Y. lipolytica*, which are located to various cellular components, participate in multiple functional processes.

### Functional enrichment analysis of acetylated proteins

**Fig. 7:**
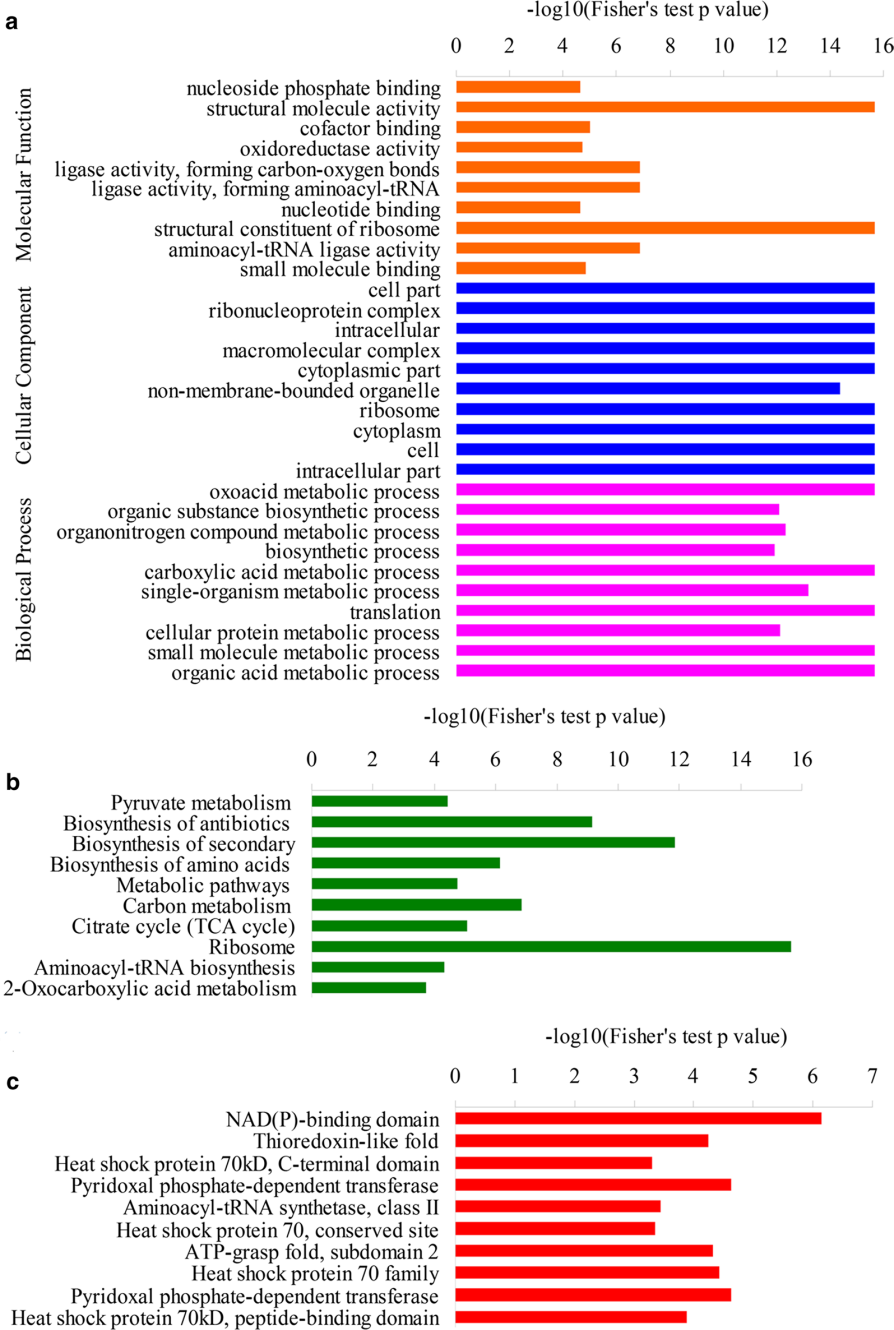
Enrichment analysis of the acetylated proteins in *Y. lipolytica*. **a** GO-based enrichment analysis in terms of molecular function, cell component and biological process. **b** KEGG pathway-based enrichment analysis of the acetylated proteins. **c** Domain-based enrichment analysis of the acetylated proteins

In order to further study what types of proteins are preferred targets for lysine acetylation, we performed a series of enrichment analyses based on GO, KEGG pathway and domain, respectively. According to GO functional enrichment, proteins with the molecular functions of structural molecule activity, structural constituent of ribosome, oxidoreductase activity, ligase activity, and nucleotide binding were found to be significantly enriched (Fig. 7a; Additional file 3: Table S2), which were associated with energy and substance metabolism. Consistent with these observations, proteins involved in organic acid metabolic process, carboxylic acid metabolic process, organic substance biosynthetic process, organonitrogen compound metabolic process, and translation were more likely to be acetylated based on the biological process analysis (Fig. 7a; Additional file 4: Table S3). The enrichment analysis based on cellular component showed that proteins distributed in cytoplasm, ribosome, macromolecular complex and non-membrane-bounded organelle have high tendency to be acetylated (Fig. 7a; Additional file 5: Table S4). In agreement with the results presented above, KEGG enrichment analysis indicated that proteins involved in citrate cycle, 2-oxocarboxylic acid metabolism, pyruvate metabolism, carbon metabolism and metabolic pathways were highly enriched (Fig. 7b; Additional file 6: Table S5). Moreover, domain enrichment analysis demonstrated that a large amount of acetylated proteins containing diverse domains, such as NAD(P)-binding domain, ATP-grasp fold subdomain, pyridoxal phosphate-dependent transferase, aminoacyl-tRNA synthetase, thioredoxin-like fold, were found to be enriched (Fig. 7c; Additional file 7: Table S6). It was worthwhile to mention that ATP, NADPH and organic acid were essential materials for fatty acid biosynthesis. These findings suggest that multiple pathways, including intracellular fatty acid metabolism, may be strictly regulated by lysine acetylation in oleaginous yeast.

### Acetylated proteins related to lipid metabolism

**Fig. 8:**
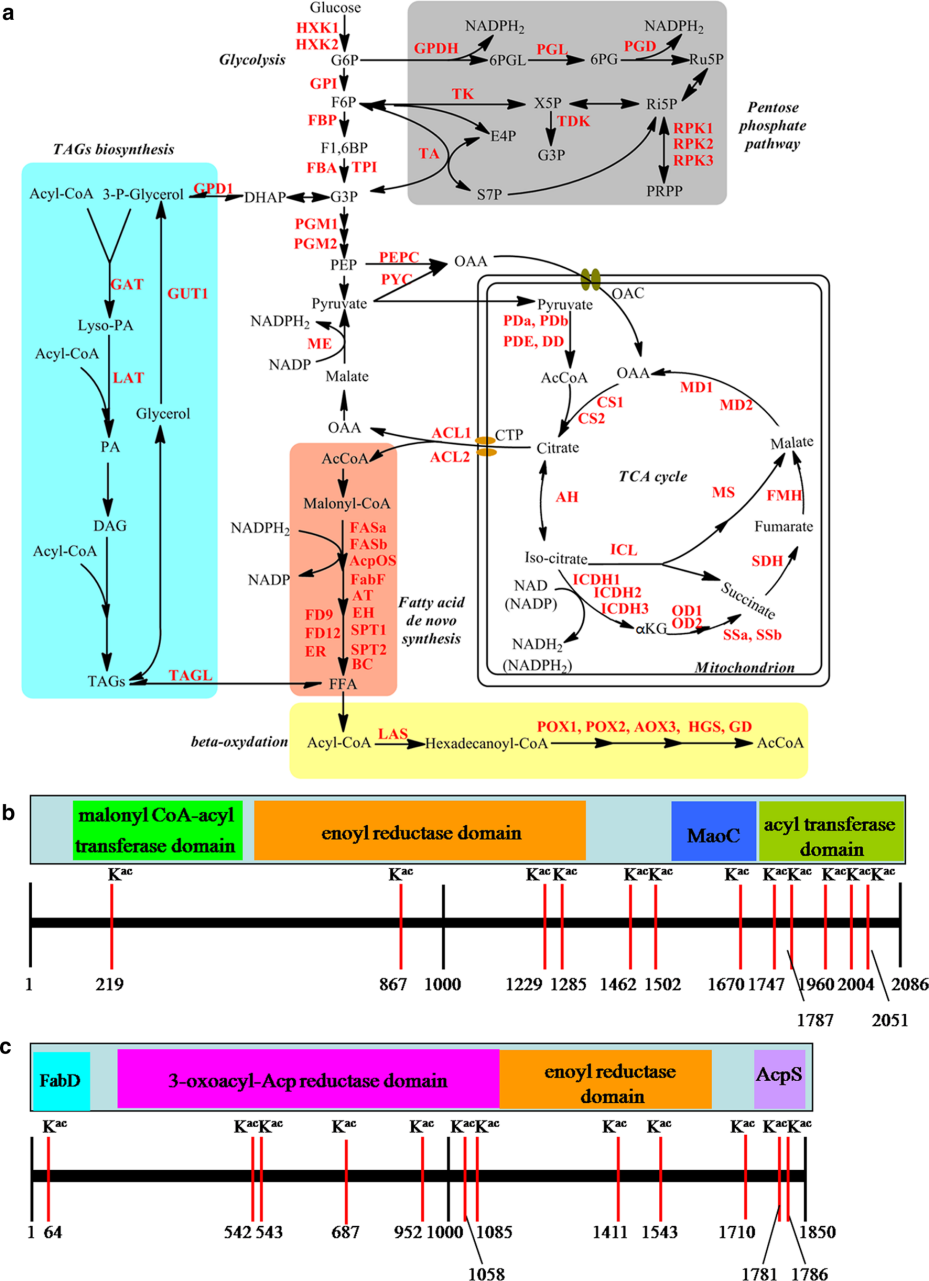
Working scheme of lysine acetylated enzymes involved in central and triacylglycerols (TAGs) metabolism pathways in oleaginous yeasts. **a** Reconstruction of central and TAGs metabolism scheme from the KEGG pathway. Identified acetylated enzymes were highlighted in* red*. Abbreviations for the components, enzyme symbols and annotations are included in Additional file 8: Tables S7. **b** An overview of acetylation sites in fatty acid synthetase (FAS1). **c** An overview of acetylation sites in fatty acid synthetase (FAS2)

Functional enrichment analysis of identified acetylated proteins revealed that intracellular lipid metabolism may be regulated by lysine acetylation in *Y. lipolytica*. To confirm these observations, we further analyzed the acetylated proteins involved in TAGs biosynthesis. Notably, a total of 65 enzymes involved in lipid biosynthesis were found to be acetylated (Fig. 8a; Additional file 8: Table S7). Our results that nearly all enzymes in major metabolic pathways, such as glycolysis, PPP, tricarboxylic acid (TCA) cycle, glyoxylate pathway, fatty acid biosynthesis and beta-oxydation are subjected to acetylation support the notion that acetylation may be linked to cellular metabolism at multiple levels.

Generally, the fatty acid chain grows through the addition of units of malonyl-CoA or acetyl-CoA, which is catalyzed by fatty acid synthetase (FAS), a multi-enzymatic complexes composed of two subunits, FAS1 (beta subunit) and FAS2 (alpha subunit) in *Y. lipolytica* (Wang et al. 2013). Interestingly, both FAS1 and FAS2 contain 12 acetylation sites in *Y. lipolytica* (Fig. 8b). Further study revealed that among these sites in FAS1, one (K219) is located in the domain of malonyl CoA-acyl carrier protein transacylase; three (K876, K1229 and K1285) are located in enoyl reductase domain of yeast-type FAS1; one (K1607) is located in the domain of MaoC; five (K1747, K1787, K2004 and K2051) are located in acyl transferase domain (Fig. 8b). In FAS2, one site (K64) in FabD domain, six sites (K542, K543, K687, K952, K1058 and K1085) in 3-oxoacyl-(acyl-carrier-protein) reductase domain, two sites (K1411, K1543) in FabB domain, and two sites (K1781, K1786) in AcpS domain, were identified (Fig. 8c). Collectively, the acetylation sites were found to be distributed in almost every types of conserved domains in the multi-enzymatic complexes of fatty acid synthetases.

### Acetylated proteins related to NADPH regeneration

NADPH generated mainly by the malic enzyme (ME) (Ratledge and Wynn 2002), is indispensable for fatty acid biosynthesis. However, another research revealed that ME was not the key enzyme for fatty acid synthesis in *Y. lipolytica*, indicating that NADPH may derive from PPP pathway and generate via a cytosolic isocitrate dehydrogenase (ICDH) (NADP^+^-dependent) (Dulermo et al. 2015). The results showed that two key enzymes in PPP pathway, glucose-6-phosphate dehydrogenase (GPDH) and 6-phosphogluconate dehydrogenase (PGD), which are responsible for generating NADPH, were both modified by acetyl groups (Fig. 8a). ICDH3, an isocitrate dehydrogenase (NADP^+^-dependent), was also found to be acetylated (Additional file 8: Table S7). Besides GPDH, PGD and ICDH3, five proteins (accession no. Q6C1K2, Q6C2T9, Q6C351, Q6CBG4 and Q6CEH4) related to NADPH regeneration were also identified to be acetylated (Additional file 4: Table S3), reflecting a crucial role of this post-translational modification in NADPH regeneration.

### Acetylated proteins involved in oxidative phosphorylation

**Fig. 9:**
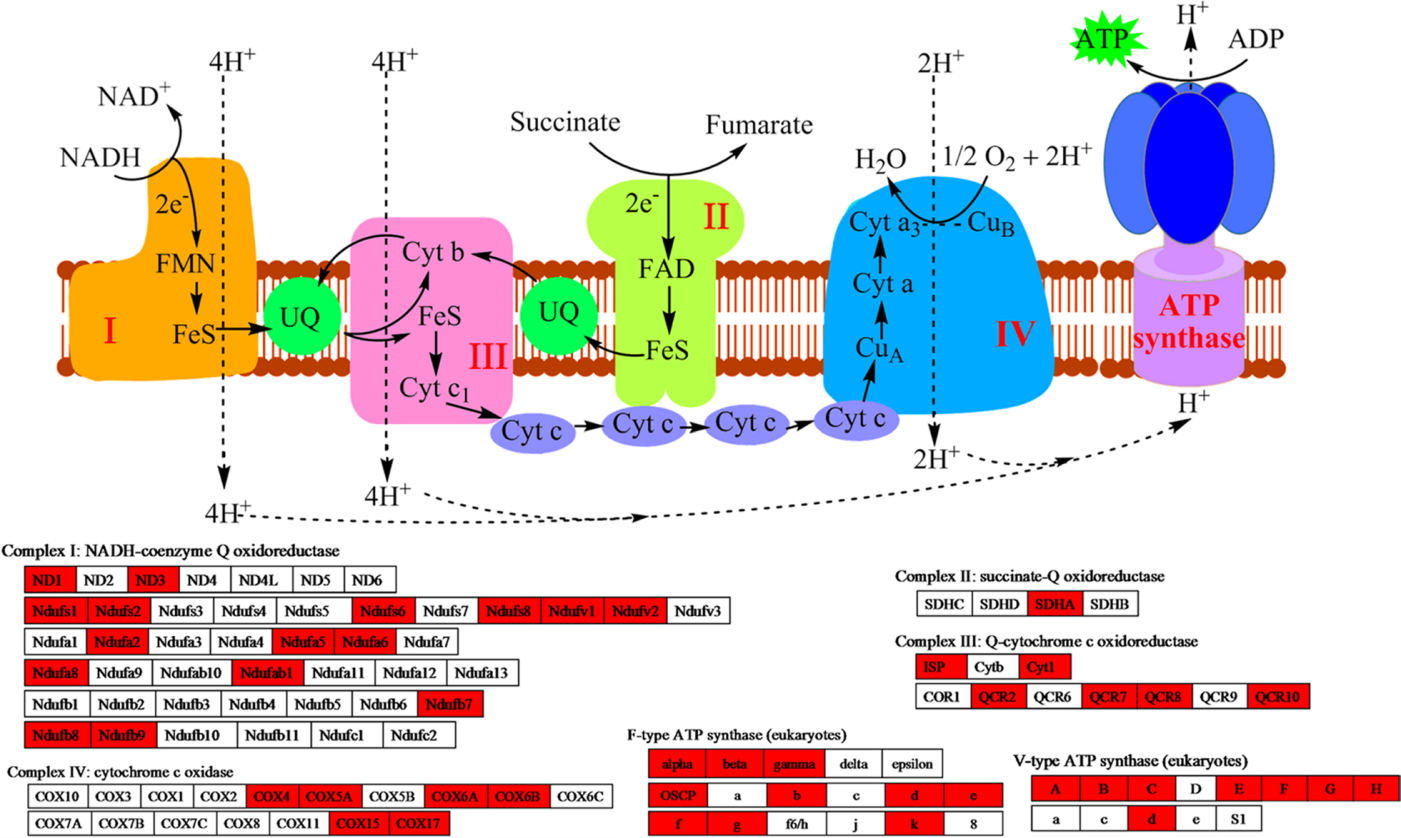
Working scheme of lysine acetylation events involved in oxidative phosphorylation. Identified acetylated proteins were highlighted in* red*

As ATP is one of the essential materials for fatty acid biosynthesis, we further investigated oxidative phosphorylation, a major biosynthesis process of ATP in *Y. lipolytica*. The results showed that four electron- transferred complexs, including NADH-coenzyme Q oxidoreductase (complex I), succinate-Q oxidoreductase (complex II), Q-cytochrome c oxidoreductase (complex III) and cytochrome c oxidase (complex IV), and ATP synthases were involved in acetylated (Fig. 9).

### Protein interaction network of acetylated proteins

**Fig. 10:**
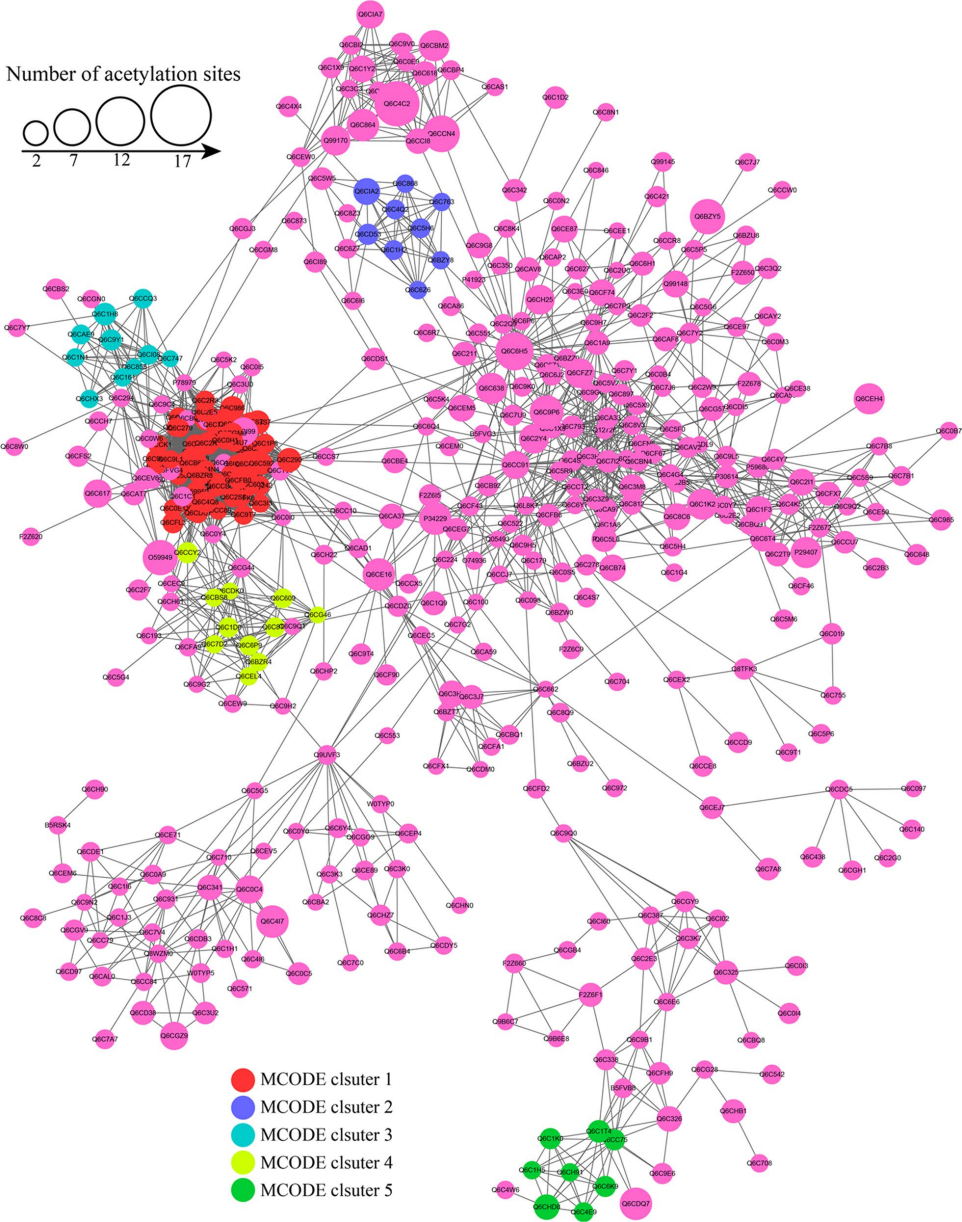
Interaction networks of the acetylated proteins in oleaginous yeast. Interaction networks of all acetylated proteins with the top five clusters of proteins associated with ribosome (*red*), aminoacyl-tRNA biosynthesis (*blue*), RNA transport (*light blue*), ribosome biogenesis in eukaryotes (*light green*) and oxidative phosphorylation (*green*)

To investigate the cellular processes regulated by acetylation in *Y. lipolytica*, we used Cytoscape software to establish the protein–protein interaction network (Fig. 10). A total of 778 acetylated proteins were mapped to the protein interaction database (Additional file 9: Table S8), which presents a global view of how acetylated proteins perform various types of functions in *Y. lipolytica*. According to the algorithm of Cytoscape software, 26 highly interconnected clusters of acetylated proteins were retrieved (Additional file 9: Table S8). The top five clusters (Cluster I–V) identified were shown in Additional file 10: Figure S2, including ribosome, aminoacyl-tRNA biosynthesis, RNA transport, ribosome biogenesis in eukaryotes and oxidative phosphorylation. The complicated interaction networks of acetylated proteins indicate that the physiological interactions among these protein complexes are likely to contribute to their cooperation and coordination in the oleaginous yeast, *Y. lipolytica*.

## Discussion

Lysine acetylation is a widespread and highly conserved post-translational modification in both eukaryotes and prokaryotes with multiple functions (Choudhary et al. 2009; Wang et al. 2010). Although many oleaginous microorganisms have been intensively investigated to produce microbial oils (Papanikolaou and Aggelis 2011; Ratledge and Wynn 2002), the regulatory mechanism of lipid biosynthesis in these organisms, especially the role of lysine acetylation in the regulation of these processes, is largely unknown. In the present study, we performed a proteomics study of lysine acetylation in the oleaginous yeast *Y. lipolytica* ACA-DC 50109.

A total of 1428 acetylated proteins with 3163 unique modification sites were identified, which is dramatically higher than those in other previously reported microorganisms (Table 1). The quantity of acetylated proteins was 1.35 times higher than that (1059) in *S. cerevisiae* (Henriksen et al. 2012), reflecting a potentially crucial role of lysine acetylation in *Y. lipolytica*. The acetylated proteins were distributed in multiple cellular compartments and participated in diverse functional biological processes, especially in lipid biosynthesis. Triacylglycerols (TAGs) is a key component of lipid droplets in oleaginous yeast (Papanikolaou and Aggelis 2011; Ratledge and Wynn 2002), which can be transformed into biodiesel by chemical ways or biological ways (Helwani et al. 2009). A large number of enzymes associated with TAGs biosynthesis were found to be acetylated (Fig. 8a), supporting the notion that lipid biosynthesis might be strictly regulated by lysine acetylation. Interestingly, two multi-enzymatic complexes of fatty acid synthetases, FAS1 and FAS2, contain 12 acetylation sites, respectively, which distribute in all the conserved domains of fatty acid synthetases.

**Table 1:** Comparison of *Y. lipolytica* acetylome with those of other published microorganisms

Species		No. of ORFs	No. of K^ac^ sites	No. of K^ac^ proteins	% of acetylation	References
Yeasts	*Y. lipolytica*	6472	3163	1428	22.1	This study
	*S. cerevisiae*	5404	2878	1059	19.6	Henriksen et al. (2012)
Fungi	*P. sojae*	19,027	2197	1150	6.0	Li et al. (2016)
	*B. cinerea*	16389	1582	954	5.82	Lv et al. (2016)
	*F. graminearum*	13,334	577	364	2.73	Zhou et al. (2016)
Bacteria	*B. amyloliquefaciens*	3811	3268	1254	32.9	Liu et al. (2016)
	*M. tuberculosis*	4034	1128	658	16.3	Xie et al. (2015)
	*Synechocystis* sp.	3672	776	513	14.0	Mo et al. (2015)
	*V. parahemolyticus*	4823	1413	656	13.6	Pan et al. (2014)
	*S. reseosporus*	6891	1143	667	9.7	Liao et al. (2014)
	*E. coli*	4144	1070	349	8.4	Zhang et al. (2013)
	*T. thermophilus*	2238	197	128	5.7	Okanishi et al. (2013)
	*P. aeruginosa*	5892	430	320	5.4	Ouidir et al. (2015)
	*S. erythraea*	7198	664	363	5.0	Huang et al. (2015)
	*B. subtilis*	4105	332	185	4.8	Kim et al. (2013)
	*S. enterica*	4430	253	191	4.3	Wang et al. (2010)
	*G. kaustophilus*	3532	253	114	3.2	Lee et al. (2013)
	*E. amylovora*	3565	142	96	2.7	Wu et al. (2013)

NADPH and ATP are indispensable for fatty acid biosynthesis (Papanikolaou and Aggelis 2011; Ratledge and Wynn 2002). In this study, eight proteins related to NADPH regeneration were found to be modified by acetyl groups (Additional file 4: Table S3), reflecting a crucial role of this post-translational modification in NADPH regeneration. Several components important for ATP synthesis, including four electron-transferred complexs and two types of ATP synthases, were identified as acetylated proteins (Fig. 9). In support of our observations, some of the electron transfer related proteins, such as ferredoxin (FD) and ferredoxin NADP^+^ reductase (FNR), were found to be lysine acetylated in cyanobacterium *Synechocystis* sp. (Mo et al. 2015). In addition, lysine acetylation was also observed on the b-subunit of chloroplastic ATP synthase in Arabidopsis (Wu et al. 2011) and rice (Xiong et al. 2016). All these findings highlight the notion that lysine acetylation might play a key regulatory role in the energy metabolic process.

To summarize, our results provide the first extensive data on lysine acetylation in oleaginous yeast *Y. lipolytica*. These data showed that lysine acetylation plays a critical regulatory role in diverse aspects of cellular process, especially in lipid biosynthesis. The dataset represents the first comprehensive view of the acetylome in oleaginous yeast and probably illuminates the crucial role of reversible acetylation in lipid biosynthesis and accumulation in oleaginous microorganisms.

## Additional files

**Additional file 1: Figure S1.** Representative MS/MS spectra of acetylpeptides from three proteins. **(a)** Acetylpeptide NLLTNFHGFDFTSDK(ac)LR with an acetylation site at K109 of the 40S ribosomal protein S1(Q6C2R9). **(b)** Acetylpeptide DK(ac)FDAAGIWYEHR with an acetylation site at K246 of the isocitrate dehydrogenase (Q6C2Y4). **(c)** Acetylpeptide DYFGAHTYQLLDGDGK(ac)WIHTNWTGR with an acetylation site at K469 of the 6-phosphogluconate dehydrogenase (Q6CEH4).

**Additional file 2: Table S1.** The identified acetylated sites in *Y. lipolytica*.

**Additional file 3: Table S2.** Protein GO enrichment based on molecular functions.

**Additional file 4: Table S3.** Protein GO enrichment based on biological process.

**Additional file 5: Table S4.** Protein GO enrichment based on cellular component.

**Additional file 6: Table S5.** Protein pathway enrichment.

**Additional file 7: Table S6.** Protein_domain_enrichment analysis.

**Additional file 8: Table S7.** Lysine acetylated enzymes related to central and lipid metabolisms in *Y. lipolytica.*

**Additional file 9: Table S8.** Protein interaction network of acetylated proteins.

**Additional file 10: Figure S2.** Interaction network of acetylated proteins associated with ribosome, aminoacyl-tRNA biosynthesis, RNA transport, ribosome biogenesis, and oxidative phosphorylation.

## Abbreviations

AGC: automatic gain control
DTT: dithiothreitol
ECL: enhanced chemiluminescence
ESI/NSI: electrospray/nanospray ionization
FA: formic acid
FAS: fatty acid synthetase
FD: ferredoxin
FDR: false discovery rate
FNR: ferredoxin NADP^+^ reductase
GO: gene ontology
GPDH: glucose-6-phosphate dehydrogenase
HATs: histone acetyltransferases
HDACs: histone deacetylases
HRP: horseradish peroxidase
IAA: iodoacetamide
KEGG: kyoto encyclopedia of genes and genomes
LC–MS/MS: liquid chromatography–mass spectrometry
ME: malic enzyme
PGD: 6-phosphogluconate dehydrogenase
PPP: pentose phosphate pathway
PTM: post-translational modification
PVDF: polyvinylidene difluoride
SDS-PAGE: sodium dodecyl sulfate polyacrylamide gel electropheresis
TAGs: triacylglycerols
TCA: tricarboxylic acid
YPD: yeast peptone dextrose
