# Supplementary material for: Systematic analysis of the lysine acetylome reveals diverse functions of lysine acetylation in the oleaginous yeast Yarrowia lipolytica
Source: AMB Express. 2017 May 12;7:94. doi: 10.1186/s13568-017-0393-2 (PMC5427063; doi:10.1186/s13568-017-0393-2)

ARTICLE

**Systematic analysis of the lysine acetylome reveals diverse functions of lysine acetylation in** **the** **oleaginous yeast *Yarrowia lipolytica***

Guangyuan Wang^1†^, Lizhong Guo^1†^, Wenxing Liang^2^ , Zhenming Chi^3^, Lin Liu^1*^

^1^College of Life Sciences, Shandong Province Key Laboratory of Applied Mycology, Qingdao Agricultural University, Qingdao 266109, China

^2^The Key Laboratory of Integrated Crop Pest Management of Shandong Province, College of Agronomy and Plant Protection, Qingdao Agricultural University, Qingdao 266109, China

^3^College of Marine Life Sciences, Ocean University of China, Qingdao 266100, China

^†^Guangyuan Wang and Lizhong Guo contributed equally to this work.

Guangyuan Wang, e-mail: gywang@qau.edu.cn

Lizhong Guo, e-mail: glz119@126.com

Lin Liu, e-mail: liulin@qau.edu.cn

Zhenming Chi, e-mail: zhenming@sdu.edu.cn

Wenxing Liang, e-mail: wliang1@qau.edu.cn

^*^**Corresponding author:**

Lin Liu, e-mail: liulin@qau.edu.cn

**Figure S1.** Representative MS/MS spectra of acetylpeptides from three proteins. **(a)** Acetylpeptide NLLTNFHGFDFTSDK(ac)LR with an acetylation site at K109 of the 40S ribosomal protein S1(Q6C2R9). **(b)** Acetylpeptide DK(ac)FDAAGIWYEHR with an acetylation site at K246 of the isocitrate dehydrogenase (Q6C2Y4). **(c)** Acetylpeptide DYFGAHTYQLLDGDGK(ac)WIHTNWTGR with an acetylation site at K469 of the 6-phosphogluconate dehydrogenase (Q6CEH4).


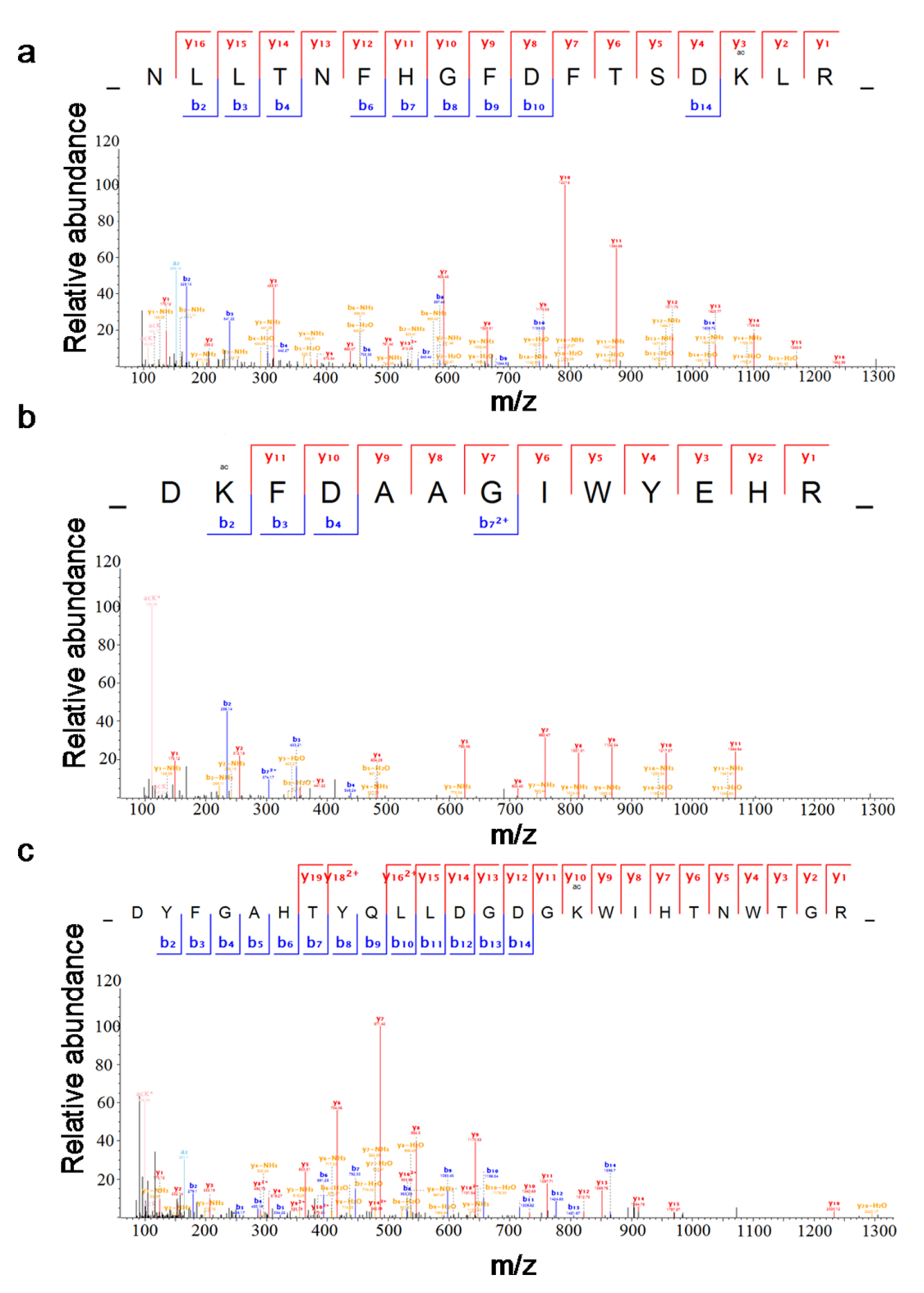

Supplement: Supplementary file 1 — Additional file 1: Figure S1. Representative MS/MS spectra of acetylpeptides from three proteins. (a) Acetylpeptide NLLTNFHGFDFTSDK(ac)LR with an acetylation site at K109 of the 40S ribosomal protein S1(Q6C2R9). (b) Acetylpeptide DK(ac)FDAAGIWYEHR with an acetylation site at K246 of the isocitrate dehydrogenase (Q6C2Y4). (c) Acetylpeptide DYFGAHTYQLLDGDGK(ac)WIHTNWTGR with an acetylation site at K469 of the 6-phosphogluconate dehydrogenase (Q6CEH4). [file 13568_2017_393_MOESM1_ESM.docx]
