# Supplementary material for: Systematic analysis of the lysine acetylome reveals diverse functions of lysine acetylation in the oleaginous yeast Yarrowia lipolytica
Source: AMB Express. 2017 May 12;7:94. doi: 10.1186/s13568-017-0393-2 (PMC5427063; doi:10.1186/s13568-017-0393-2)
Supplement: Supplementary file 8 — Additional file 8: Table S7. Lysine acetylated enzymes related to central and lipid metabolisms in Y. lipolytica. [file 13568_2017_393_MOESM8_ESM.doc]

**Table S7. Lysine acetylated enzymes related to central and lipid metabolisms in *Y.lipolytica***

| **Enzymes** | **Entry** | **Annotation** | **Positions** |
| --- | --- | --- | --- |
| ACL1 | YALI0E34793p | ATP citrate lyase subunit 1 | 304, 522, 608 |
| ACL2 | YALI0D24431p | ATP citrate lyase subunit 2 | 100, 109, 134, 257, |
| AcpOS | YALI0F30679p | 3-oxoacyl-[acyl-carrier-protein] synthase II | 216 |
| AH | YALI0D09361p | aconitate hydratase | 45, 46, 75, 89, 159, 410, 514, 568, 586, 590, 604, 688, 766 |
| AOX3 | YALI0D24750p | acyl-coenzyme A oxidase 3 | 102, 402, |
| AT | YALI0B08536p | acetyl-CoA C-acetyltransferase | 132, 183, 221, 270, 274, |
| BC | YALI0C11407p | acetyl-CoA carboxylase / biotin carboxylase 1 | 55, 88, 536, 624, 1014, 1311, 1800, 2072, 2172 |
| CS1 | YALI0E00638p | citrate synthase | 37, 312, 316, 322,456 |
| CS2 | YALI0E02684p | citrate synthase | 102, 148, 337, 450, 456 |
| DD | YALI0D20768p | dihydrolipoamide dehydrogenase | 95, 135, 138, 143, 259,406, 421, 486, |
| EH | YALI0B10406p | enoyl-CoA hydratase | 90, 222, 329 |
| ER | YALI0C19624p | trans-2-enoyl-CoA reductase | 235, 239, 259, 295, 310, 324 |
| FabF | YALI0D00385p | fabF:3-oxoacyl-[acyl-carrier-protein] synthase II | 424 |
| FASa | YALI0B19382p | fatty acid synthase subunit alpha, | 64, 542, 543, 687, 952, 1058, 1085, 1411, 1543, 1710, 1781, 1786 |
| FASb | YALI0B15059p | fatty acid synthase subunit beta | 219, 867, 1229, 1285, 1462, 1502, 1670, 1747, 1787, 1960, 2004, 2051, |
| FBA | YALI0E26004p | fructose-bisphosphate aldolase, class II | 115, 236, 244, 361 |
| FBP | YALI0A15972p | fructose-1,6-bisphosphatase I | 242 |
| FD12 | YALI0B10153p | Oleate delta-12 desaturase | 179 |
| FD9 | YALI0C05951p | Delta-9 fatty acid desaturase | 117, 156 |
| FMH | YALI0C06776p | fumarate hydratase, class II | 32, 87 |
| GAT | YALI0C00209p | glycerol-3-phosphate O-acyltransferase / dihydroxyacetone phosphate acyltransferase | 160, 383 |
| GD | YALI0F23749p | Glutaryl-CoA dehydrogenase | 310 |
| GPD1 | YALI0B13970p | Glycerol-3-phosphate dehydrogenase | 120, 207, 209, 446, |
| GPDH | YALI0E22649p | glucose-6-phosphate 1-dehydrogenase | 27, 68, 69, 100, 146, 154, |
| GPI | YALI0F07711p | Glucose-6-phosphate isomerase | 27, 38, 150, 243, 250, 314, 317, 381 |
| GUT1 | YALI0F00484p | Glycerol kinase | 151, 210,216, 465, 489 |
| HGS | YALI0F30481p | hydroxymethylglutaryl-CoA synthase | 68, 300, 380 |
| HXK1 | YALI0B22308p | hexokinase | 96,320,453 |
| HXK2 | YALI0E15488p | hexokinase | 165, 302 |
| ICHD1 | YALI0D06303p | isocitrate dehydrogenase (NAD+) | 53, 204, 209,352, |
| ICHD2 | YALI0E05137p | isocitrate dehydrogenase (NAD+) | 35, 192,203, 208, 332 |
| ICHD3 | YALI0F04095p | isocitrate dehydrogenase | 33, 75, 93, 99, 232, 241, 244, 246, 362, 383, |
| ICL | YALI0F31999p | isocitrate lyase | 216 |
| LAS | YALI0D17864p | long-chain acyl-CoA synthetase | 50, 54, 82,109, 113, 291, 511, 631, |
| LAT | YALI0E18964p | lysophosphatidate acyltransferase | 95 |
| MD1 | YALI0D16753p | malate dehydrogenase | 163, 307, 314 |
| MD2 | YALI0E14190p | malate dehydrogenase | 143, 201, 307 |
| ME | YALI0E18634p | malate dehydrogenase (oxaloacetate-decarboxylating) | 35, 149, 457, 483, |
| MS | YALI0E15708p | malate synthase | 158, 297, 491, |
| OD1 | YALI0E33517p | 2-oxoglutarate dehydrogenase E1 component | 233, 375, 555, 562, 624, 846, 995, |
| OD2 | YALI0E16929p | 2-oxoglutarate dehydrogenase E2 component (dihydrolipoamide succinyltransferase) | 260, 265, 340 |
| PDa | YALI0F20702p | pyruvate dehydrogenase E1 component alpha subunit | 245, 271, 393 |
| PDb | YALI0E27005p | pyruvate dehydrogenase E1 component beta subunit | 44, 156, 219, 226, |
| PDE | YALI0D23683p | pyruvate dehydrogenase E2 component (dihydrolipoamide acetyltransferase) | 75, 258, 326, 422 |
| PEPC | YALI0C16995p | phosphoenolpyruvate carboxykinase (ATP) | 469, 532, 540 |
| PGD | YALI0B15598p | 6-phosphogluconate dehydrogenase | 45, 73, 162, 170, 247, 259, 308, 323, 384, 407, 469 |
| PGL | YALI0C19085p | 6-phosphogluconolactonase | 286 |
| PGM1 | YALI0B02728p | 2,3-bisphosphoglycerate-dependent phosphoglycerate mutase | 3, 17, 80, 129, 148, 191, |
| PGM2 | YALI0D09229p | probable phosphoglycerate mutase | 100, 150 |
| POX1 | YALI0E18568p | 3-ketoacyl-CoA thiolase, peroxisomal | 198 |
| POX2 | YALI0E11099p | Peroxisomal acetoacetyl-CoA thiolase | 235, 93 |
| PYC | YALI0C24101p | pyruvate carboxylase | 82, 191, 308, 1036 |
| RPK1 | YALI0B00836p | ribose-phosphate pyrophosphokinase | 112 |
| RPK2 | YALI0B13552p | ribose-phosphate pyrophosphokinase | 163 |
| RPK3 | YALI0E32351p | ribose-phosphate pyrophosphokinase | 19 |
| SDH | YALI0D11374p | succinate dehydrogenase (ubiquinone) flavoprotein subunit | 168, 598 |
| SPT1 | YALI0C03179p | serine palmitoyltransferase | 437 |
| SPT2 | YALI0F15345p | serine palmitoyltransferase | 10, 21, 249 |
| SSa | YALI0E24013p | succinyl-CoA synthetase alpha subunit | 38,141, 316,324 |
| SSb | YALI0D04741p | succinyl-CoA synthetase beta subunit | 111, 115, 217, 417, 278, |
| TA | YALI0F15587p | transaldolase | 10, 52, 107, 122, 146, 190, 195, 221, 271, 278, 317 |
| TAGL | YALI0D19184p | triacylglycerol lipase | 103 |
| TDK | YALI0F09273p | triose/dihydroxyacetone kinase / FAD-AMP lyase (cyclizing) | 33, 281, 374, 487 |
| TK | YALI0E06479p | transketolase | 7, 275, 301, 319, 326, 369, 513 |
| TPI | YALI0F01584p | triosephosphate isomerase | 331 |
