# Supplementary material for: Systematic analysis of the lysine acetylome reveals diverse functions of lysine acetylation in the oleaginous yeast Yarrowia lipolytica
Source: AMB Express. 2017 May 12;7:94. doi: 10.1186/s13568-017-0393-2 (PMC5427063; doi:10.1186/s13568-017-0393-2)
Supplement: Supplementary file 10 — Additional file 10: Figure S2. Interaction network of acetylated proteins associated with ribosome, aminoacyl-tRNA biosynthesis, RNA transport, ribosome biogenesis, and oxidative phosphorylation. [file 13568_2017_393_MOESM10_ESM.docx]

**Figure S2.** Interaction network of acetylated proteins associated with ribosome, aminoacyl-tRNA biosynthesis, RNA transport, ribosome biognenesis, and oxidative phosphorylation.

**
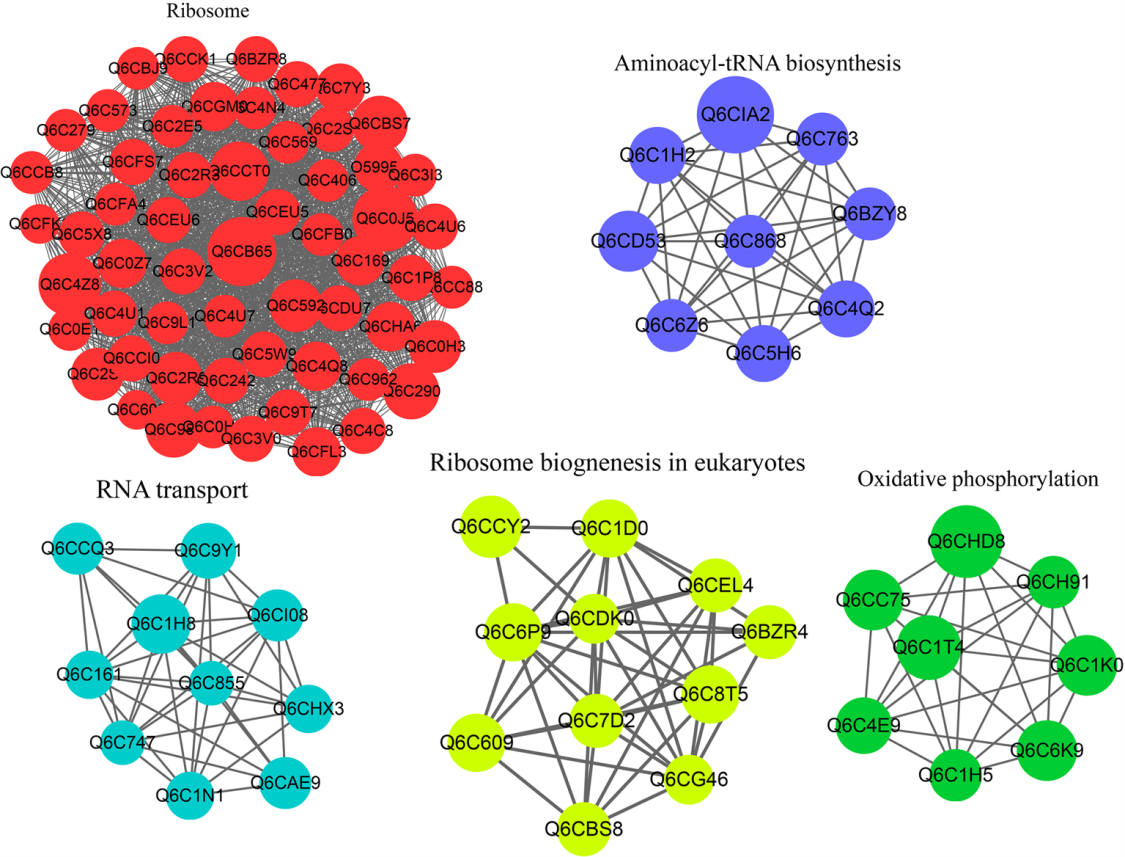
**
